# Adjuvant Effects of Health Education of Chinese Medicine for Chronic Diseases: A Systematic Review and Meta-Analysis of Randomized Controlled Trials

**DOI:** 10.1155/2020/3738753

**Published:** 2020-03-31

**Authors:** An-Lu Wang, He Zhang, Jie Zhang, Yan Zhang, Hui-Juan Cao, Jian-Ping Liu, Hao Xu, Ke-Ji Chen

**Affiliations:** ^1^Beijing University of Chinese Medicine, Beijing, China; ^2^Xiyuan Hospital, China Academy of Chinese Medical Sciences, Beijing, China

## Abstract

**Objective:**

To evaluate the adjuvant effects of health education of Chinese medicine (HECM) for patients with three types of common noncommunicable diseases (NCD-hypertension, diabetes, and coronary heart disease (CHD)).

**Methods:**

The protocol of this review was registered in the PROSPERO website (CRD42017058325). Six databases were searched till Sep. 30, 2019. Randomized controlled trials (RCTs) comparing HECM plus conventional therapy with conventional therapy were retrieved. Participants were diagnosed as one of the 3 above NCDs. HECM is regarded as lectures and classes about diet therapy, exercise therapy, emotion balance, and other knowledge according to Chinese medicine theory. The control rate of the disease was defined as a primary outcome in this review. Outcomes were synthesized using meta-analyses where reporting was sufficiently homogeneous or alternatively synthesized in a systematic review.

**Results:**

In total, 12 trials with 1142 patients were included in this review. Since all the trials may have unclear or high risk of bias, only low quality evidence could be found for supporting the adjunctive effect of HECM in treating hypertension, diabetes, and CHD, to reduce the control rate (risk ratio −1.58), the blood pressure level (mean difference −9.38 mmHg), the fasting plasma glucose level (mean difference −1.26 mmol/L), and the symptoms of angina.

**Conclusion:**

The adjunctive effect of HECM on increasing the control rate of hypertension, improving the symptoms of diabetes and CHD, was only supported by low-quality evidence in this review. More rigorous trials with larger sample sizes and higher quality are warranted to provide a high quality of evidence.

## 1. Introduction

Noncommunicable diseases (NCDs) are chronic diseases of long duration and generally slow progression. In the MESH database (https://www.ncbi.nlm.nih.gov/mesh/), NCDs are defined as diseases which are typically noninfectious in origin and do not transmit from an affected individual to others. The four main types of NCDs are cardiovascular (e.g., heart attacks and stroke), cancer, chronic respiratory diseases (e.g., chronic obstructive pulmonary disease and asthma), and diabetes mellitus. It is reported that NCDs killed almost 40 million people annually, with nearly 70% of all deaths globally [1]. Cumulative economic losses of US $7 trillion will be paid, and millions of people will be trapped in poverty over the next 15 years caused by the heavy burden from NCDs [2]. NCDs affect people in low- and middle-income countries more. In China, NCDs have become the leading causes of mortality and caused heavy economic burden as well. Cardiovascular diseases, hypertension, and diabetes mellitus caused millions of Chinese people's death each year [3, 4]. As we all know, with the changing of lifestyle and diets, the risk factors of NCDs are increased among the Chinese population, such as tobacco use, overuse of alcohol, physical inactivity, and unhealthy diet. Furthermore, metabolic risk factors, including raised blood pressure, overweight, hyperglycemia, and hyperlipidemia, increase the risk of NCDs.

Therefore, besides the adequate treatments for lessening the impact of NCDs, it is important to focus on reducing the risk factors that cause NCDs. In the community population, the disease control of most NCDs patients (e.g., hypertension, diabetes mellitus, and coronary heart disease) relies on self-management, but the disease control rate (less than 50% for hypertension or diabetes) does not meet the expectations of health management departments. Thus, carrying out health education for community NCDs patients has become an attempted health service in China. Health education generally contains the education of rational drug usage, regular consultation, psychological supports, and lifestyle guidance. Evidence from systematic reviews showed that plenty of treatments of Chinese medicine, such as acupuncture, moxibustion, Tai Chi, and herbal medicine, may have clinical effects in treating specific NCDs [5–9]. Moreover, substantial trials suggested that health education of Chinese medicine (HECM) may be an effective complementary intervention when treating NCDs by controlling the risk factors of related diseases [10–15]. However, no systematic research evidence summarized the effectiveness of HECM. Therefore, we conduct this systematic review and meta-analysis to evaluate the adjuvant effects of HECM on the basis of conventional therapy in treating three common types of NCDs: hypertension, diabetes, and coronary heart disease (CHD).

## 2. Methods

The protocol of this review was registered in the PROSPERO website, which cited Wang Anlu, Cao Huijuan, and Liu Jianping. Chinese medicine health education for common noncommunicable diseases in community: a systematic review and meta-analysis of randomized controlled trials. PROSPERO 2017 CRD42017058325 is available at http://www.crd.york.ac.uk/PROSPERO/display_record.php?ID=CRD42017058325.

### 2.1. Inclusion Criteria

Eligible studies should be randomized controlled trials (RCTs) regardless of the language and publication status; the study setting should be located in the community and should meet the following criteria: (1) participants should be diagnosed as one of the three types of NCDs (hypertension, diabetes, or CHD) according to a recognized criterion. (2) Health education of Chinese medicine (HECM), such as lectures and classes about any life-style relevant knowledge based on Chinese medicine theory, as an adjunctive therapy of basic treatment (e.g., medications) for the specific NCDs is regarded as the intervention. The contents of HECM included diet education, mood balance education, exercise education, life style education according to Chinese medicine theory, and popularization of basic disease knowledge. Education was defined as a learning process in which doctors assisted patients in learning the contents of HECM instead of treating them directly. (3) Control should be the same basic treatment as in the HECM group; the basic treatment included standard treatment or conventional therapy for the specific NCDs. (4) The primary outcome included the rehospitalization rate; all-cause mortality; control rate or key symptoms improvement (e.g., blood pressure for patients with hypertension, blood glucose and/or hemoglobin A1C (HbA1C) for patients with diabetes, and recurrence of cardiovascular events for patients with CHD). The secondary outcomes include other symptoms improvement, such as heart rate, cardiac function measured by echocardiogram, diabetes complication, blood lipid, patients' compliance of treatment, status of depression or anxiety measured by recognized scale (such as Hamilton Depression/Anxiety Scale), and patients' satisfactory for education/treatment.

### 2.2. Literature Search Strategy

We searched the following databases from their inception to 30 September 2019: PUBMED, Cochrane Central Register of controlled trials, Chinese Scientific Journal Database, China National Knowledge Infrastructure (CNKI), and Wanfang Database and Sino-Med Database. The searching strategy was used as follows:

  1# “Chinese medicine” OR “traditional Chinese medicine”
  2# “health education” OR “health promotion” OR “education”
  3# “hypertension” OR “essential hypertension” OR “blood pressure”
  4# “diabetes” OR “diabetes mellitus” OR “blood sugar” OR “blood glucose”
  5# “coronary heart disease” OR “coronary artery disease” OR “angina” OR “angina pectoris” OR “myocardial infarction”
  6# “randomized controlled trial” OR “RCT”
  7# 1# AND 2# AND 3# AND 6#
  8# 1# AND 2# AND 4# AND 6#
  9# 1# AND 2# AND 5# AND 6#
  10# 7# OR 8# OR 9#

We also carefully scanned the references of all the eligible articles of RCTs to identify further publications.

### 2.3. Data Extraction and Quality Assessment

Retrieved studied were reviewed by two reviewers (H. Zhang and Y. Zhang) independently to screen the eligible trials according to the above criteria. Data of the included studies were then extracted based on a standard data-collection form, including first author name, publication year, regions, patient characteristics (sample size, gender, and age), methods of therapy, and outcomes. Differences between these two reviewers were discussed and resolved seriously. Another two reviewers (A. L. Wang and H. J. Cao) assessed the methodological quality of RCTs by using the Cochrane risk of bias tool (ROB) [16] independently. Disagreements were solved by consensus or consulting the third author (J. P. Liu). The ROB standard covers the following items: random sequence generation, allocation concealment, blinding of participants and personnel, blinding of outcome assessment, incomplete outcome data, selective reporting, and other bias. For each item, it can be assessed as low, high, or unclear risk of bias.

### 2.4. Statistical Analysis

RevMan 5.3 software provided by Cochrane Collaboration was used for data analyses. We calculated mean difference (MD) with corresponding 95% confidence interval (CI) for continuous outcomes and risk ratio (RR) with its 95% CI for dichotomous data. Quantitative meta-analysis was performed when the trials had similar characteristics regarding types of participants, intervention, comparison, outcomes measurements, and the statistical heterogeneity among trials was acceptable (the *I*^2^ is less than 75%). If necessary, we would conduct the subgroup analysis according to the age/gender of patients, the type of HECM, and the duration of the education. Publication bias would be assessed by funnel plot if data permitted.

### 2.5. Evidence Evaluation

The Grades of Recommendations Assessment, Development and Evaluation (GRADE) [17] was used to assess the quality of the evidence for each primary outcome with meta-analysis. Considering the following aspects, such as methodological quality, outcome consistency of trials, directness, and accuracy of evidence and possibility of publication bias, we judged whether to degrade the evidence of included trials and assessed the level of the evidence as high, moderate, low, or very low.

## 3. Results

### 3.1. Study Selection and Characteristics

**Figure 1:**
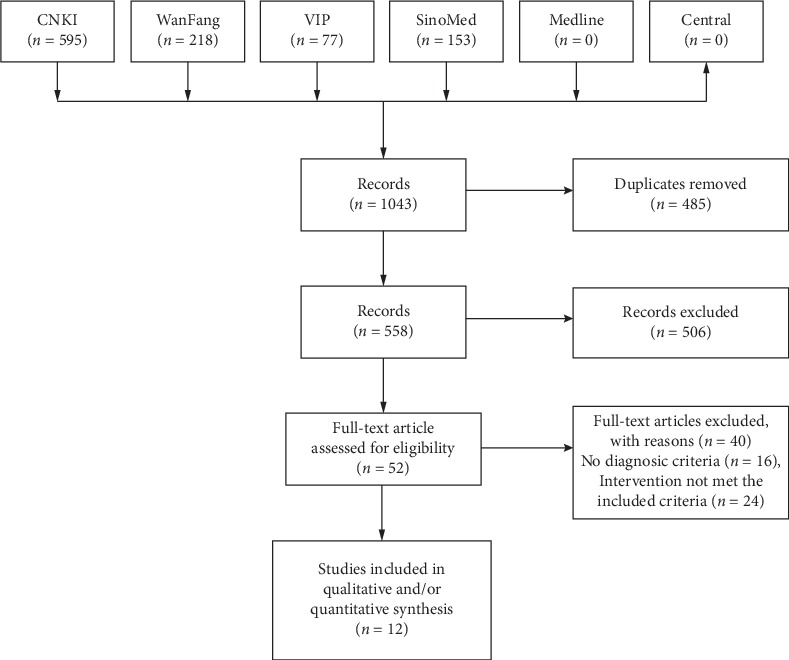
Study flow chart.

The initial search retrieved 1122 articles from the six databases. After removing the duplicates, 618 trials were identified. Through screening the titles and abstracts, 60 trials remained. Moreover, 12 trials [18–29] with 13 articles [18–30] were included finally after reading the full-text (Figure 1).

The publication year of the included trials ranged from 2013 to 2019. All the trials were conducted in mainland China. In total, 1142 patients were included in these 12 trials. All trials were conducted in the community and more than 58.86% of participants were male. Six trials [18–23] focused on hypertensive patients, five trials [24–28] concerned diabetic patients, and one trial [29] included patients with coronary heart disease. Nine trials [19–25, 27, 29] reported the average duration of diseases, which range from 5 to 19 years.

All studies compared HECM plus routine western drugs to the drugs alone. HECM included lectures and classes about diet therapy, exercising training, emotion balance, massage guidance, manipulation (tuina) guidance and/or lavipeditum (zuyu) guidance based on Chinese medicine theory. Three articles [20, 21, 24] reported the frequency of the education, which was once a week.

The hypertension-related outcomes included control rate [18, 21–23], level of blood pressure [19–23] (including systolic blood pressure (SBP) and diastolic blood pressure (DBP)), disease awareness [19, 21], and compliance of treatment [21]. The diabetes-related outcomes included fasting plasma glucose (FPG) [24–28], HbA1c [25], disease awareness [25], and self-care activity [26, 28]. The CHD-related outcomes included scores of Seattle angina questionnaire, symptoms of angina, and the consumption of nitroglycerin tablets [29] (details of the characteristics of the included trials are shown in Table 1).

### 3.2. Methodological Quality

**Figure 2:**
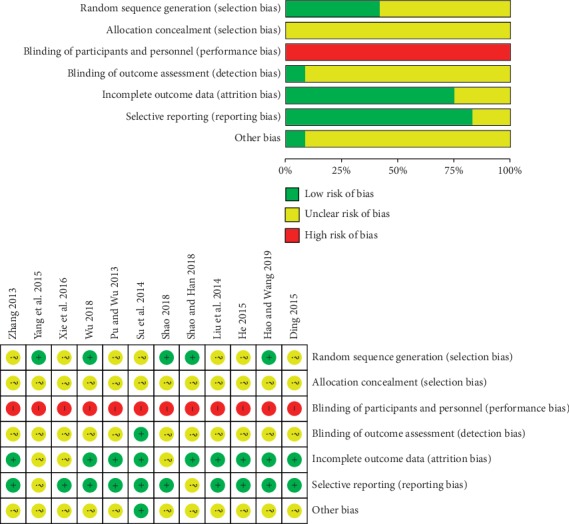
Risk of bias graph and summary of included studies.

Since the majority of included trials reported insufficient information to judge whether the method was likely to introduce bias, we assessed most of them as having an unclear risk of bias. Only five trials [24, 26–29] reported the methods of random sequence generation, which were assessed as having low risk of selection bias. However, none of them mentioned the allocation concealment methods. Performance bias is of high risk due to no study ever using placebo control or other methods to blind participants and personnel. Detection bias, attrition bias, reporting bias, and other biases were hard to determine due to the limited information provided in the reports. All trials reported neither dropout nor sample size calculation. Over all, the methodological quality of the included trials was not promising (Figure 2).

### 3.3. Effects of HECM

No trial reported the rehospitalization rate and all-cause mortality for any of the NCD.

#### 3.3.1. For Patients with Hypertension

**Figure 3:**
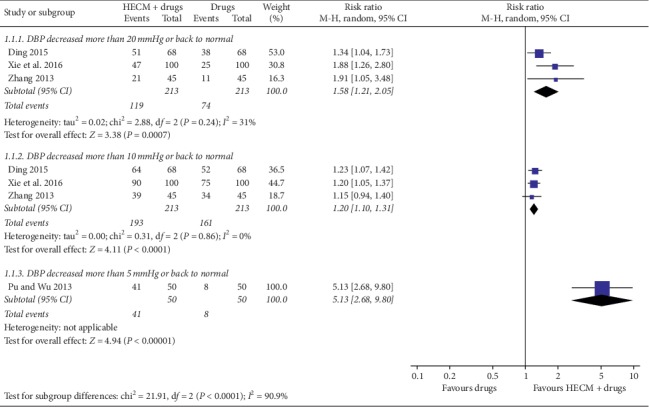
Forest plot of comparison of HECM plus drugs vs. drugs for hypertension on control rate SBP/DBP.

*(1) Control Rate of Hypertension*. Three trials [18, 22, 23] reported the control rate which counted by the proportion of number of patients whose blood pressure level back to normal (DBP less than 90 mmHg) or DBP decreased more than 20 mmHg (RR = 1.58, 95% CI 1.21 to 2.05, *n* = 426, *I*^2^ = 31%); one trial [21] also reported the control rate of hypertension, which is defined as number of patients whose blood pressure level returned back to normal or DBP decreased more than 5 mmHg (RR = 5.13, 95% CI 2.68 to 9.80, *n* = 100). We conducted subgroup meta-analysis for these four trials according to the definition of control (DBP decreased more than 20 mmHg, 10 mmHg, or 5 mmHg); all subgroups showed better effect of HECM as adjunctive therapy for increasing the control rate of hypertension (Figure 3). However, there was obvious statistical heterogeneity among the subgroups (*I*^2^ = 90.9%).

**Figure 4:**
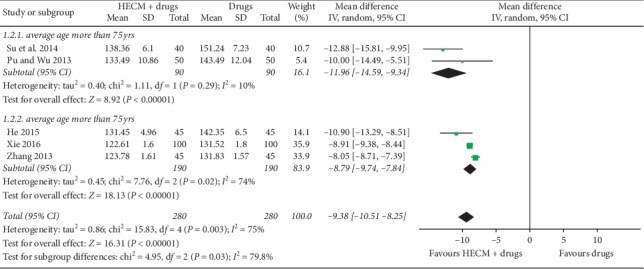
Forest plot of comparison of HECM plus drugs vs. drugs for hypertension on SBP.

**Figure 5:**
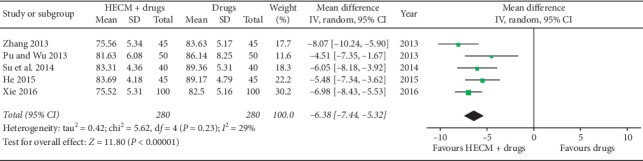
Forest plot of comparison of HECM plus drugs vs. drugs for hypertension on DBP quality of life.

Five trials [19–23] reported the level of SBP and DBP posttreatment. Meta-analysis showed significantly better effect of HECM combined with drugs for decreasing the level of SBP (MD = −9.38 mmHg, 95% CI −10.51 to −8.25 mmHg, *n* = 560, *I*^2^ = 75%, Figure 4) and DBP (MD = −6.38 mmHg, 95% CI −7.44 to −5.32 mmHg, *I*^2^ = 29%, *n* = 560, Figure 5). Considering the obvious heterogeneity among trials, a subgroup meta-analysis was conducted according to patients' age for the level of SBP. It seems larger estimate effects are more likely to be found in elder patients (average age more than 75 years old) concerned about decreasing level of SBP.

One trial [19] reported quality of life assessed by The Short Form Health Survey 36 (SF-36), which showed HECM group was superior to control group in four sections of SF-36, including physical functioning (MD = −2.53, 95% CI −4.15 to −0.91, *n* = 90), social role functioning (MD = 0.57, 95% CI 0.05 to 1.09, *n* = 90), bodily pain (MD = −1.17, 95% CI −1.91 to −0.43, *n* = 90), and emotional role functioning (MD = 0.85, 95% CI 0.48 to 1.22, *n* = 90).

*(2) Disease Awareness*. Two trials [19, 21] reported hypertension-related questionnaire scores pre- and posttreatment. One trial [19] assessed patients knowledge for disease etiology and risk factors (RR = 1.72, 95% CI 1.34 to 2.20, *n* = 90), clinical symptoms (RR = 1.54, 95% CI 1.24, to 1.92, *n* = 90), and treatment (RR = 2.33, 95% CI 1.66 to 3.27, *n* = 90) by counting the proportion of patients with good knowledge in those fields. The other trial [21] measured the patients' knowledge by scores of hypertension-related knowledge questionnaire (MD = 52.90, 95% CI 47.79 to 58.01, *n* = 100).

*(3) Patients Compliance*. One trial [21] reported patients' compliance to the medication and follow up, the result showed HECM may statistically have an effect on increasing the numbers of participants who adherence to the doctors' advice and regularly take drugs (RR = 1.52, 95% CI 1.11 to 2.09, *n* = 100).

#### 3.3.2. For Patients with Diabetes

**Figure 6:**
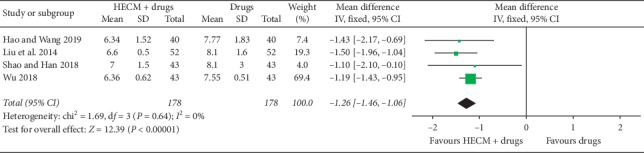
Forest plot of comparison of HECM plus drugs vs. drugs for diabetes on fast plasma glucose 2 h postprandial plasma glucose.

*(1) FPG Level*. Five trials [24–28] reported this outcome, which showed HECM combined with drugs was statistically more effective than drugs alone in decreasing the FPG level (MD = −1.26 mmol/L, 95% CI −1.46 to −1.06 mmol/L, *n* = 356, *I*^2^ = 0%, Figure 6).

**Figure 7:**
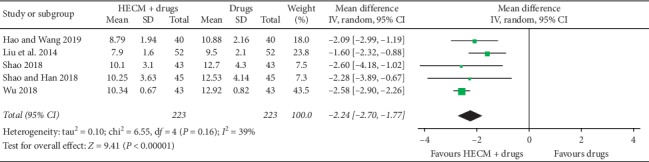
Forest plot of comparison of HECM plus drugs vs. drugs for diabetes on 2 h postprandial plasma glucose.

The meta-analysis of 5 trials [24–28] showed good add-on effect of HECM based on drugs (MD = −2.24 mmol/L, 95% CI −2.70 to −1.77 mmol/L, *n* = 104, *I*^2^ = 39%, Figure 7) for controlling the 2 h postprandial plasma glucose.

*(2) HbA1c Level*. One trial [25] reported HbA1c level at the 6^th^ month, which showed that a combination group was statistically more effective than the control for this outcome (MD = −0.30%, 95% CI −0.40 to −0.20%, *n* = 104).

*(3) Self-Control Activity*. Two trials [26, 28] assessed the patients' self-control activity after intervention. One of them reported the proportion of patients who showed good self-management behavior on appropriate exercise (RR = 1.42, 95% CI 1.04 to 1.93, *n* = 86), diet control (RR = 1.36, 95% CI 1.01 to 1.83, *n* = 86), blood sugar monitor (RR = 1.37, 95% CI 1.06 to 1.78, *n* = 86), and regular medication (RR = 1.31, 95% CI 1.04 to 1.66, *n* = 86) between groups. Another one reported similar outcomes but were assessed by the reliability of the diabetes care profile (DCP). HECM combined with drugs also showed better effect on increasing the patients self-control ability concerning appropriate exercise (MD = 0.38, 95% CI 0.26 to 0.50, *n* = 86), diet control (MD = 1.17, 95% CI 1.11 to 1.23, *n* = 86), blood sugar monitor (MD = 0.94, 95% CI 0.73 to 1.15, *n* = 86), and regular medication (MD = 1.23, 95% CI 1.12 to 1.34, *n* = 86).

*(4) Patients Compliance*. One trial [25] evaluated the patients' compliance according to the self-designed questionnaire. Of the total 50 scores, HECM may help in increasing the patients' compliance compared to the drugs group (MD = 6.60, 95% CI 5.07 to 8.13, *n* = 104).

#### 3.3.3. For Patients with Coronary Heart Disease

*(1) Seattle Angina Questionnaire (SAQ)*. The only one study [29] (with 61 cases) concerning stable angina pectoris measured this outcome. Whether the total scores of SAQ (MD = 4.90, 95% CI 0.57 to 9.23) or each of the five dimensions of coronary artery disease all showed the better effect of HECM in adjunctive with drugs. The five dimensions included physical limitation (MD = 8.37, 95% CI 3.81 to 12.93), angina stability (MD = 8.37, 95% CI 3.81 to 12.93), angina frequency (MD = 3.93, 95% CI 0.98 to 6.88), treatment satisfaction (MD = 4.25, 95% CI 0.86 to 7.64), and disease perception (MD = 7.74, 95% CI 4.50 to 10.98).

*(2) The Dosage of Nitroglycerin Tablets*. HECM group was superior to control on decreasing the monthly consumption of nitroglycerin tablets (MD = −5.52 mg, 95% CI −10.14 to −0.90 mg, *n* = 61).

### 3.4. Additional Analysis

Due to the limited number of included studies in one meta-analysis, the publication bias cannot be assessed by funnel plot. Meanwhile, no trials reported safety-related outcomes.

## 4. Discussion

### 4.1. Main Findings from the Review

Twelve trials with 1142 patients are included in this review. The meta-analysis showed a potential good adjunctive effect of HECM for the specific NCDs. For hypertension, HECM combined with drugs seemed to gain 21% more patients whose blood pressure level was back to normal (lower than 90 mmHg). Meanwhile, combined with HCEM, drugs may decrease 9.38 mmHg more SBP or 6.38 mmHg more DBP after at least 3 months of treatment. For diabetes, HECM also was effective as an adjunctive therapy on controlling FPG level (MD = −1.26 mmol/L), 2 h postprandial plasma glucose (MD = −2.24 mmol/L), and HbA1c level (MD = −30%) compared to drugs. For CHD, HECM seems to have more advantages in improving the symptoms of angina, to lower 4.9 scores of SAQ, and to reduce 5.52 mg monthly consumption of nitroglycerin tablets. Though all the included trials did not mention any safety relevant outcomes, some of them reported that HECM may improve the patients' compliance and awareness of the disease.

### 4.2. Overall Quality of the Evidence

Due to the unclear or high risk of selection bias, performance bias, detection bias, and/or other bias for the majority of the included trials, the evidence for all outcomes should be downgraded for two levels according to GRADE assessment. Besides, the sample size of the included trials was limited (less than 200 participants in meta-analysis with continuous outcomes or less than 300 events in meta-analysis with dichotomous outcomes), which lead to one level downgraded for imprecision. Publication bias was also suspected even though the funnel plot could not be done since all the studies were published in China with limited included participants, low quality, and all reported positive results to support the HECM application. Thus, only “very low” quality evidence could be provided to affirm the adjunctive effect of HECM for all the relevant outcomes in treating patients with hypertension, diabetes, or CHD. Summary of findings for the primary outcomes of the three specific NCDs is shown in Tables 2–4.

### 4.3. Reflections on the Effective Mechanism

The overall “very low” quality evidence showed adjunctive with HECM may increase 21% more patients whose blood pressure level was back to normal or at least decreased 20 mmHg DBP after 3 months of medication treatment. It is very interesting when we looked through the results of the subgroup analysis shown in Figure 3. These four trials [19, 20, 22, 23] reported not only the control rate but also the numbers of patients whose DBP decreased at least 10 mmHg in both groups, and the estimate value showed HECM group may have 15% more patients achieved this therapeutic effect. Compared to the first subgroup of control rate, it prompts that after 3 months of medication treatment, 75% of patients' DBP level may have more than 10 mmHg reduction, but only 35% of patients' DBP may have more than 20 mmHg reduction. However, for patients who were over 75 years old, only 16% of them may get more than 5 mmHg reduction of DBP after medication treatment [21]. Subgroup analysis of posttreatment SBP level also found similar results that elder patients (over 75 years old) may achieve more SBP reduction (MD = −11.96 mmHg, 95% CI −14.59 to −9.34 mmHg) than younger patients (MD = −8.79 mmHg, 95% CI −9.74 to −7.84 mmHg) under HECM (see Figure 4). Despite the difference of drugs and other study application characteristics among those trials, it may point out that elder patients had poor self-administration ability and compliance to doctors' advice. Thus, health education may be more helpful for them to regularly use drugs, pay attention to the risk factors of diseases, and promote self-health behavior management.

In this review, the contents of HECM are composed by education of diet control, exercise, or other nonpharmaceutical therapy according to TCM theory and emotional balance. Since we did not compare the difference between HECM and other types of health education, we considered that partially or even large proportion of the effectiveness of HECM may be caused by the education itself. Recent research studies suggest that the improvements of SBP, FPG, BMI, smoking, and physical inactivity may reduce the premature mortality of NCDs [31]. However, despite joint efforts by public health and medical professionals in the last decade, awareness, treatment, and control of NCDs remain poor. Only 40.9% of hypertensive individuals aware of their condition and only 9.7% of them controlled their blood pressure well (SBP ≤ 140 mmHg and DBP ≤ 90 mmHg) [32]. Therefore, health education is necessary for community patients with NCDs.

### 4.4. Implications for Clinical Practice and Future Research

According to the main findings and the quality of the evidence, the average blood pressure level of hypertension patients who accepted HECM was well controlled, and the average FPG level of diabetic patients was controlled as well (less than 7 mmol/L). Thus, we suggest that community medical institutions should regularly carry out health education for patients with NCDs. The duration of the education should be at least 3 months, and the content of the education should contain medication guidance and general knowledge of disease prevention and treatment. The knowledge of health preservation and protection related to TCM can be educated according to patients' acceptance or operability.

The current very low quality of evidence is mainly due to the high risk of bias of the included studies; thus, we suggest researchers design and conduct high quality randomized controlled trials to testify the effectiveness of HECM in the future. To clarify the specific effectiveness of HECM, the control group could be non-TCM relevant health education. Sample size calculation should be done during the trial design period. Age of the patients could be considered as a stratification factor of randomization since we found the potential influence of age for the final outcomes. If the future study intends to compare the HECM and common type of health education, the outcome of health economics should also be included for cost-effectiveness analysis.

## 5. Conclusion

HECM, especially health education, seems to have advantages as an adjunctive therapy on improving the effectiveness of NCDs, including hypertension, diabetes, and CHD. However, due to the small sample size and potential bias of most included trials, this result should be interpreted with caution. More high-quality trials concerning long-term effects are warranted before the strong recommendation of HECM as a complementary therapy for NCDs.

## Figures and Tables

**Table 1:** Characteristics of the 12 included trials.

Study ID	% male	Sample size	Average age (year) (range)		Treatment course (month)	Outcomes
			Intervention	Control		
*For patients with hypertension*						
Ding 2015 [18]	53.68	136	49.54 ± 5.63	50.87 ± 5.96	6	Control rate
He 2015 [19]	56.67	90	57.1 ± 11.4 (45–79)	56.8 ± 11.2 (41–80)	12	SBP/DBP, hypertension knowledge level, SF-36
Su et al. 2014 [20]	50.00	80	77.94 ± 0.53 (75–80)	76.32 ± 0.65 (74–80)	3	SBP/DBP
Wu 2013 [21]	50.00	100	75.5 ± 4.7	74.8 ± 5.3	3	Control rate, SBP/DBP, hypertension-related questionnaire, compliance of patients
Xie et al. 2016 [22]	58.50	200	59.67 ± 6.13 (45–78)	60.37 ± 5.19 (43–80)	3	SBP/DBP, control rate
Zhang 2013 [23]	62.22	90	62.4 (42–80)	65.1 (39–81)	9	SBP/DBP, control rate

*For patients with diabetes*						
Hao and Wang 2019 [24]	67.50	80	66.1 ± 3.1 (55–79)	63.6 ± 4.2 (53–76)	NR	Fasting glucose, 2 h postprandial plasma glucose, diabetic knowledge level, SF-36
Liu et al. 2014 [25]	55.77	104	44–81		6	Fasting glucose, 2 h postprandial plasma glucose, HbA1c, compliance of patients
Shao and Han 2018 [26]	52.33	86	27–71	29–70	NR	Fasting glucose, 2 h postprandial plasma glucose, diabetic self-care activity
Shao 2018 [27]	58.89	90	53.14 ± 5.52 (38–73)	54.42 ± 5.37 (37–75)	NR	Fasting glucose, 2 h postprandial plasma glucose
Wu 2018 [28]	50.00	86	54.57 ± 3.26 (46–63)	54.68 ± 3.48 (47–64)	12	Fasting glucose, 2 h postprandial plasma glucose, diabetic self-care activity, TG, TC

*For patients with coronary heart disease*						
Yang et al. 2015 [29]	52.46	61	58 ± 6.33 (41–73)	57 ± 7.12 (43–74)	6	SAQ, the dosage of nitroglycerin tablets

NR: not reported; SBP: systolic blood pressure level; DBP: diastolic blood pressure level; TG: total cholesterol; TC: triglyceride; SAQ: Seattle Angina Questionnaire.

**Table 2:** Summary of findings for patients with hypertension.

HECM + drugs compared to drugs for diabetes					
Patient or population: diabetes					
Setting: community					
Intervention: HECM + drugs					
Comparison: drugs					
Outcome No. of participants (studies)	Relative effect (95% CI)	Anticipated absolute effects (95% CI)			Certainty
		Control	HECM^∗^	Difference	
Control rate: DBP decreased more than 20 mmHg or back to normal assessed with blood pressure level No. of participants: 426 (3 RCTs)	RR 1.58 (1.21 to 2.05)	34.7%	54.9% (42 to 71.2)	20.2% more (7.3 more to 36.5 more)	⊕○○○ Very low^a,b,c,d^
Systolic blood pressure level (SBP) No. of participants: 560 (5 RCTs)	—	The mean systolic blood pressure level was **135.91** mmHg	—	MD **9.38** mmHg lower (10.51 lower to 8.25 lower)	⊕○○○ Very low^a,b,d^
Diastolic blood pressure level (DBP) No. of participants: 560 (5 RCTs)	—	The mean diastolic blood pressure level was **85.86** mmHg	—	MD **6.38** mmHg lower (7.44 lower to 5.32 lower)	⊕○○○ Very low^a,d^
GRADE working group grades of evidence. High certainty: we are very confident that the true effect lies close to that of the estimate of the effect.					
Moderate certainty: we are moderately confident in the effect estimate. The true effect is likely to be close to the estimate of the effect, but there is a possibility that it is substantially different.					
Low certainty: our confidence in the effect estimate is limited. The true effect may be substantially different from the estimate of the effect.					
Very low certainty: we have very little confidence in the effect estimate. The true effect is likely to be substantially different from the estimate of effect.					
Explanations					
(a) Most of the trials had an unclear risk of selection bias, detective bias, or other bias, all of them had a high risk of performance bias.					
(b) There was potential statistical heterogeneity among trials (*I*-square value >50%).					
(c) Number of events less than 300.					
(d) All the included trials published in China with positive results and a small sample size.					

^*∗*^The risk in the intervention group (and its 95% confidence interval) is based on the assumed risk in the comparison group and the relative effect of the intervention (and its 95% CI). CI: confidence interval; RR: risk ratio; MD: mean difference.

**Table 3:** Summary of findings for patients with diabetes.

HECM + drugs compared to drugs for diabetes					
Patient or population: diabetes					
Setting: community					
Intervention: HECM + drugs					
Comparison: drugs					
Outcome No. of participants (studies)	Relative effect (95% CI)	Anticipated absolute effects (95% CI)			Certainty
		Control	HECM	Difference	
Fast glucose (FPG) No. of participants: 356 (4 RCTs)	—	The mean fast glucose was **7.70** mmol/L	—	MD **1.26** mmol/L lower (1.46 lower to 1.06 lower)	⊕○○○ Very low^a,b^
2 h postprandial plasma glucose No. of participants: 446 (5 RCTs)	—	The mean 2 h postprandial plasma glucose was **11.71** mmol/L	—	MD **2.24** mmol/L lower (2.7 lower to 1.77 lower)	⊕○○○ Very low^a,b,c^
GRADE working group grades of evidence					
High certainty: we are very confident that the true effect lies close to that of the estimate of the effect.					
Moderate certainty: we are moderately confident in the effect estimate. The true effect is likely to be close to the estimate of the effect, but there is a possibility that it is substantially different.					
Low certainty: our confidence in the effect estimate is limited. The true effect may be substantially different from the estimate of the effect.					
Very low certainty: we have very little confidence in the effect estimate. The true effect is likely to be substantially different from the estimate of effect.					
Explanations					
(a) Most of the trials had an unclear risk of selection bias, detective bias, and other bias, all of them had a high risk of performance bias.					
(b) All of the trials published in China with positive results and small sample size.					
(c) There was potential statistical heterogeneity among trials (*I*-square value more than 40%).					

^*∗*^The risk in the intervention group (and its 95% confidence interval) is based on the assumed risk in the comparison group and the relative effect of the intervention (and its 95% CI). CI: confidence interval; MD: mean difference.

**Table 4:** Summary of findings for patients with coronary heart disease.

HECM plus drugs compared to drugs for stable angina					
Patient or population: stable angina					
Setting: community					
Intervention: HECM plus drugs					
Comparison: drugs					
Outcome No. of participants (studies)	Relative effect (95% CI)	Anticipated absolute effects (95% CI)			Certainty
		Control	HECM^∗^	Difference	
Seattle Angina Questionnaire (SAQ) No. of participants: 61 (1 RCT)	—	The mean seattle Angina Questionnaire was **72.77** scores	—	MD **4.9** scores higher (0.57 higher to 9.23 higher)	⊕○○○ Very low^a,b,c^
Nitroglycerin Tablets consumption No. of participants: 61 (1 RCT)	—	The mean nitroglycerin tablets consumption was **45.87** mg	—	MD **5.52 **mg lower (10.14 lower to 0.9 lower)	⊕○○○ Very low^a,b,c^
GRADE working group grades of evidence					
High certainty: we are very confident that the true effect lies close to that of the estimate of the effect.					
Moderate certainty: we are moderately confident in the effect estimate. The true effect is likely to be close to the estimate of the effect, but there is a possibility that it is substantially different.					
Low certainty: our confidence in the effect estimate is limited. The true effect may be substantially different from the estimate of the effect.					
Very low certainty: we have very little confidence in the effect estimate. The true effect is likely to be substantially different from the estimate of effect.					
Explanations					
(a) The trial had an unclear risk of selection bias, detective bias, and other bias also had a high risk of performance bias.					
(b) Number of cases less than 200.					
(c) Only one trial included, which was published in China and had a small sample size.					

^*∗*^The risk in the intervention group (and its 95% confidence interval) is based on the assumed risk in the comparison group and the **relative effect** of the intervention (and its 95% CI). CI: confidence interval; MD: mean difference.

## Additional Points

*Practical Implications*. Community medical institutions could regularly carry out health education for patients with NCDs. The duration of the education should be at least 3 months, and the content of the education should contain medication guidance and general knowledge of disease prevention and treatment.
